# Variation in clinical decision-making for induction of labour: a qualitative study

**DOI:** 10.1186/s12884-017-1518-y

**Published:** 2017-09-22

**Authors:** Tanya A. Nippita, Maree Porter, Sean K. Seeho, Jonathan M. Morris, Christine L. Roberts

**Affiliations:** 1 Clinical and Population Perinatal Health Research, Kolling Institute of Medical Research, Northern Sydney Local Health District, Level 5, Douglas Building, Royal North Shore Hospital, St Leonards, NSW, 2065 Australia; 2 Sydney Medical School-Northern, University of Sydney, St Leonards, NSW 2065 Australia; 3 Department of Obstetrics and Gynaecology, Royal North Shore Hospital, Northern Sydney Local Health District, St Leonards, NSW 2065 Australia

**Keywords:** Labour induction, Obstetrics, Decision-making, Doctor-patient relationship, Variation, Risk assessment

## Abstract

**Background:**

Unexplained variation in induction of labour (IOL) rates exist between hospitals, even after accounting for casemix and hospital differences. We aimed to explore factors that influence clinical decision-making for IOL that may be contributing to the variation in IOL rates between hospitals.

**Methods:**

We undertook a qualitative study involving semi-structured, audio-recorded interviews with obstetricians and midwives. Using purposive sampling, participants known to have diverse opinions on IOL were selected from ten Australian maternity hospitals (based on differences in hospital IOL rate, size, location and case-mix complexities). Transcripts were indexed, coded, and analysed using the Framework Approach to identify main themes and subthemes.

**Results:**

Forty-five participants were interviewed (21 midwives, 24 obstetric medical staff). Variations in decision-making for IOL were based on the obstetrician’s perception of medical risk in the pregnancy (influenced by the obstetrician’s personality and knowledge), their care relationship with the woman, how they involved the woman in decision-making, and resource availability. The role of a ‘gatekeeper’ in the procedural aspects of arranging an IOL also influenced decision-making. There was wide variation in the clinical decision-making practices of obstetricians and less accountability for decision-making in hospitals with a high IOL rate, with the converse occurring in hospitals with low IOL rates.

**Conclusion:**

Improved communication, standardised risk assessment and accountability for IOL offer potential for reducing variation in hospital IOL rates.

## Background

Variation in rates of medical intervention has occurred for decades [1]. Although variation in interventions occurs due to differences in population co-morbidities and patient preference, it is unexplained variation that is of concern. Unexplained variation in rates of intervention raises doubt about the appropriateness of the intervention, the efficient use of health care resources [2] and may be due to differences in physician practice styles and resource allocation [1].

One of the commonest interventions in pregnancy is induction of labour (IOL), which is associated with almost one-quarter of all births, [3, 4] with wide unexplained variation in rates of IOL between countries, [5] provinces [6] and hospitals, despite adjusting for differences in patient comorbidities [7, 8]. In obstetrics, constraints and differences in scheduling contributed to variation in geographical practice patterns for caesarean section [9]. Sabastiao and colleagues recommend further qualitative investigation to understand the contribution of communication and shared decision-making between obstetric providers and women for obstetrical interventions such as caesareans [9]. Women’s experiences and preferences for IOL have been explored, [10–12] but there are no studies investigating the role of physician practice styles, preferences and decision-making to explain variation in hospital IOL rates. Therefore, we aimed to explore factors that influence clinical decision-making for IOL in New South Wales (NSW), Australia using qualitative research methods.

## Methods

Employing a Framework Approach, we undertook a qualitative study using semi-structured interviews with obstetricians and midwives. Using stratified purposive sampling, 10 hospital sites were selected from all NSW maternity hospitals based on previous work [13]. NSW is the most populous state in Australia, comprising 7.5 million people and one-third of all Australian births. Hospitals were selected based on differences in their rates of IOL for nulliparae at term as these rates showed the most variation [9] (five hospitals with high IOL rates and five hospitals with low IOL rates); location (urban, rural); size (small: <500 births per annum; medium: 500–2000 births per annum; large: >2000 births per annum); type of care (public, private); and obstetric case-mix complexities (tertiary, district and private). Public models of care include obstetrician-based care, shared-care (obstetrician/midwifery care or General Practitioner/midwifery care), and midwifery-based care. The private model of maternity care involves the woman choosing a specific obstetrician to care for her throughout the antenatal, intrapartum and postnatal periods.

A senior member of staff (midwife or obstetrician, as advised by the head of the department) at each hospital was asked to identify potential participants and inform them about the study. Obstetricians, trainee obstetricians and midwifery staff with diverse opinions on IOL practice and decision-making, and awareness of different practices within the hospital were invited to participate in an interview.

Following written, informed consent, semi-structured individual private interviews were conducted face-to-face by the researcher (TN) between February and July 2015 at a time and location convenient to the participant (usually their workplace office). The interview participants knew the occupation (obstetrician) and current workplace (none of the ten hospitals) of the researcher (TN). The researcher (TN) had a prior professional collegial relationship with six of the participants and had previously worked at one of the hospitals three years prior to the interviews. These professional relationships facilitated the interview process and interest in participating in the study.

Quantitative findings [13] from a previous study guided the initial development of interview questions. Interview questions were designed to elicit demographic information, participant’s decision-making regarding IOL and examples of decision-making for IOL. To test, refine and develop questions, pilot interviews were conducted with two clinicians working at hospitals not included in the study. Interviews were conducted until data saturation (within and between hospitals) was reached. All interviews were audio-recorded, transcribed verbatim and de-identified. The researcher (TN) clarified and checked responses by email or phone conversations with participants. No participant requested their interview be removed from analysis and no repeat interviews were performed. Brief field notes were made after each interview to assist with contextualizing the data. In the study, participants were identified by the hospital code, participant number and the clinical group, with ‘MW = midwife’ and ‘O = obstetrician/obstetric registrar/GP obstetrician’ (Hospital code_participant number_clinical group i.e. G_P30_MW: ‘G’ = Hospital G; ‘P30’ = Participant 30; ‘MW’ = Midwife).

NVivo 10 (QSR International, Doncaster Australia) was used for systematic and interconnected data indexing, coding, development of the framework matrix and analysis of the interview transcripts. Two investigators (TN, MP) separately reviewed all transcript data for themes. The first five transcripts were analysed using five initial deductive themes developed from interview topics. Thematic data were then compared and discussed to identify additional emerging themes. Inductive coding then followed using codes established from emerging themes. Two clinical researchers (TN, SS) then coded all transcripts using this coding framework, with further refinement of main themes and subthemes.

Ethical approval to conduct the study was granted by the Northern Sydney Local Health District Human Research Ethics Committee (LNR/15/HAWKE/1).

## Results

Forty-five participants were interviewed: 21 midwives (nine midwifery unit managers, 12 senior midwives) and 24 obstetric medical staff (16 consultant obstetricians, four trainee obstetricians, four GP-obstetricians), with the interviews lasting a median of 31 min (range: 17 to 62 min). Participants had worked in their current hospital for a median of 11 years (range: 3 months to 35 years). The characteristics of the hospitals are displayed in Table 1.

**Table 1:** Characteristics of the sample hospitals

Hospital	Size of hospital^a^	Geographic location	Type of care^b^	IOL rate for nulliparae at term (%) [13]	Hospital rate for IOL
A	Small	Urban	Public district	11	Low
B	Small	Rural	Public district	11	Low
C	Medium	Rural	Public district	11	Low
D	Large	Urban	Private	12	Low
E	Medium	Urban	Public district	13	Low
F	Medium	Urban	Public district	25	High
G	Large	Urban	Public tertiary	27	High
H	Large	Urban	Private	28	High
I	Large	Urban	Public tertiary	29	High
J	Small	Rural	Public district	30	High

^a^Large hospitals > 2000 births per annum; medium hospitals 500–2000 births per annum; small hospitals < 500 births per annum

^b^Type of care: Public care- a range of models of care depending on need including obstetric care, mixed obstetric/midwifery care or midwifery led care (district care- birth and care for mothers and babies with normal and moderate risk factors; tertiary care- care for mothers and babies with normal, moderate and high risk factors); Private care- private obstetrician led care

**Fig. 1:**
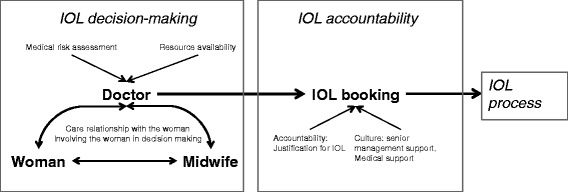
Conceptual mode of the factors determining decision making for IOL

Qualitative analysis of transcript and field note data found recurring themes reflecting variation in IOL decision-making of participants. Participants reported decision-making for IOL occurred predominantly between the woman and their obstetrician, with some influence from the midwife if they were involved in the woman’s antenatal care. Across hospitals, participants reported an obstetrician made the final decision to perform an IOL and arranged this through a person who maintained a register of induction bookings for the hospital (usually the delivery suite manager). The conceptual model between IOL decision-making, the IOL booking and the IOL process is illustrated in Fig. 1.

Four themes emerged in data analysis that reflected variation in participants’ IOL decision-making: IOL decision-making using medical risk assessment, IOL decision-making influenced by the care relationship with the woman, involving the woman in decision-making and IOL decision-making influenced by resource availability.

### Theme 1: IOL decision-making using medical risk assessment

In this study, risk assessment was an obstetrician’s perceived risk of an adverse event for a woman and/or her baby by continuing the pregnancy compared with the risks associated with IOL. Obstetricians were particularly concerned about perinatal death and medical litigation:

‘...*if there are any adverse outcomes, well that's - you know it's all the obstetrician's fault, so - so I tend to do inductions very easily’* (H_P21_O).

‘...*there is somewhere in the back of their [obstetricians’] mind, litigation, definitely. I think they’re more aware of patients wanting to perhaps sue later on if,* “*why didn't you do this [IOL] if you knew it was a risk?”*’ (B_P30_MW).

Perceived obstetrician’s personality and knowledge were identified as subthemes influencing IOL decision-making.

#### Personality

Participants reported that an obstetrician’s personality contributed to differences in risk assessment, with the ‘relaxed’ obstetrician having a lower rate of IOL compared with the ‘anxious’ obstetrician:

*‘...if I look at the people that don’t have a high induction rate, um, they’re probably, uh, the kind of obstetricians who are quite relaxed in their practice, and not the type to get stressed.’* (D_P16_MW).

#### Knowledge

Clinical knowledge from the medical literature, clinical practice guidelines and personal experience also shaped obstetric risk assessment. Obstetricians stated that experiential knowledge and situations that led to adverse birth outcomes influenced their practice more than guidelines or the medical literature:

*‘...the problem I have with overall hospital protocols is that they – and also absolute insistence on evidence-based research – is it takes the art out of medicine in the general sense of – that you get after doing it for a long period of time’* (D_P09_O).

‘*Some consultants and some midwives are a little bit more anxious, so they might induce closer to 38 [weeks gestation] when I might have sat on somewhere to 39 [weeks gestation]. But that’s because last year they sat on someone to 39 [weeks gestation] and it didn’t go as well.’* (I_P41_O).

Obstetricians expressed that the evidence on the risks and benefits of IOL to guide decision-making was lacking, especially around the likelihood of vaginal birth compared with caesarean section after IOL:

‘...*consultants probably have different opinions about it [IOL], probably because there is no clear evidence which pathway to go*.’ (E_P28_O).

Clinical assessment and investigations such as ultrasound played a major role in obtaining information to assess risk in the pregnancy. There was general agreement that particular risk factors such as gestational diabetes and pre-eclampsia warranted IOL, but there was variation and uncertainty over the timing of the IOL. Occasionally, ultrasound appeared to increase uncertainty as to whether a woman should have an IOL. A hospital with a high rate of IOL organised third trimester ultrasounds for all women, and one participant queried the evidence to support this investigation. Participants in hospitals with high and low rates of IOL questioned the reliability of ultrasound:

‘...*we in [this hospital] have a bit of difficulty...we get abnormal Dopplers that are then normal when they’re rechecked....we refer them down to [another hospital] and they don’t detect the same problem’* (C_P46_O).

The consequences for rural women with an abnormal ultrasound report were often significant and included the need to travel several hours to the closest city for a second opinion ultrasound. Clinicians were often placed in a situation in which they had to either trust the ultrasound and offer IOL or request a second opinion ultrasound and closely monitor the fetus.

### Theme 2: IOL decision-making influenced by the care relationship with the pregnant woman

The medical and midwifery staff’s relationship with the woman, thereby knowing and understanding the woman’s wishes and desires, played a large role in IOL decision-making. Obstetricians and midwives described the benefits especially in an antenatal care model with one health care practitioner seeing that woman (continuity of care):

*‘I know what she’s like, I know what’s important to her...so therefore her wishes and her desires and her feelings about how she’s going in the pregnancy play a bigger part in any decision-making process*’ (F_P03_O).

However, other participants felt the close relationship developed through a continuity of care model resulted in the health care practitioner being:

*‘...more attached, and then they [health care practitioner] can’t take an objective decision; it becomes subjective’* (F_P2_O).

The relationship between the health care provider and woman could also make it challenging for obstetricians to refuse a request for IOL. Obstetricians and midwives acknowledged this difficulty, describing it as much easier to acquiesce to a woman’s request for IOL, especially in the private sector:

‘...*induction is seen as part of the service in that your job as an obstetrician is to induce me because that's what you do because I paid your money, you're going to do something*’ (H_P17_O).

### Theme 3: involving the woman in IOL decision-making

Participants were uncertain whether women had autonomy over decision-making for IOL, describing how they influenced the woman’s decision depending on their assessment of need or agreement for IOL:

*...‘it depends on how you put it to the patient as to how much you scare them...when you see that there is no good reason [for IOL], and you think that you’re better off waiting, well, then you stress the, the bad things a bit more*.’ (B_P32_O).

Participants were also doubtful of women’s understanding of the process and risks of IOL and discussed the need to improve antenatal education and provide realistic expectations of birth and labour:

‘*I think there's a community perception out there that it's [IOL] relatively safe, relatively easy and they can just do it, you know, without really understanding all the consequences. They actually think it's risk free*’ (B_P30_MW).

The limitations of paper-based information were also recognised and participants felt that time constraints in the clinic reduced the provision of information:

‘*I think everybody wants to provide it [education]. I think it’s about the ability to provide it. If you’re given 10 min for an antenatal visit with a woman, you’ve got Buckley’s [no chance] of imparting as much information as you’d like in that 10 min*’ (I_P40_MW).

### Theme 4: IOL decision-making influenced by resource availability

Medical, midwifery and other health professional availability was a factor in decision-making for IOL across hospitals with differing geographical locations, type of care (public/private) and maternity service capabilities. Medical practitioners who had consultation rooms geographically away from the hospital, or those that had deliveries at multiple locations would plan IOL when they were physically located close to the hospital, to ensure their availability if an emergency was to occur:

*‘I'm in this hospital 2 days a week, Wednesdays, Thursdays. I try and do - do my inductions on those days...so I'm available all the time’* (H_P21_O).

Medical availability of the obstetrician and ancillary medical staff was a recurring theme that influenced IOL decision-making in rural areas. GP obstetricians described how their main work was in their consulting rooms, with deliveries during office hours being disruptive for themselves, patients and other ancillary medical staff (such as GP anaesthetists) if an emergency operative delivery was required. Therefore, planned deliveries in the form of IOL would help manage and minimise disruption to consulting room workloads:

*‘They [the obstetrician] will choose their time to suit themselves...So often they’ll induce on a day that’s suitable for their workload.’ (F_P04_MW)*

Midwifery resource limitations and the ability to accommodate labouring women limited availability of IOL, yet this was predominantly a rural issue. A midwife manager discussed the reduction in the number of IOL that can be offered based on concerns about patient safety:

‘....the *town is only reasonably small, with only a certain number of midwives, and if I've used all those midwives, there's no-one else*’ (B_P30_MW).

### Differences between hospitals with high and low IOL rates

Two themes appeared to differentiate hospitals with high and low rates of IOL, but these were not consistent across all hospitals. High IOL hospitals mostly demonstrated greater variation in the clinical decision-making practices of obstetricians within that hospital and less accountability for that decision, with the converse true for low IOL hospitals.

### Theme 5: variation in IOL decision-making practices among obstetricians within hospitals

Hospitals that had wide variation in obstetricians’ decision-making philosophies and practices were more often hospitals with high rates of IOL. If a hospital had one obstetrician that tended to induce women compared to their colleagues, women were able to ‘doctor shop’ within the hospital and have the IOL that the previous obstetrician had refused.

In contrast, hospitals with low rates of IOL tended to have obstetricians with similar decision-making philosophies; one obstetrician at a hospital with a low rate of IOL commented:

‘*... with [the other obstetrician] and I being there, um, I think things [rates of IOL] are .... better, because our views [on IOL] are similar, we don’t interfere unless we feel there’s a good need*.’ (B_P32_O).

## Theme 6: accountability for IOL decision-making

The main mechanism for accountability appeared to be the way an IOL was arranged at the hospital, and the influence of a ‘gatekeeper’ (for the purposes of this study defined as the person or people who organise the specific date for a woman’s IOL, as requested by the obstetrician or the midwife). The booking for IOL was usually in a paper-based diary format. For most hospitals, the midwifery manager or the senior midwife on duty held the book and was responsible for booking IOL. In contrast, in some hospitals the gatekeeper was seen as a purely administrative role, with administrative or clerical staff processing IOL requests from obstetricians and midwives.

When a senior midwife or the midwifery manager with the authority to question the decision for IOL played the role of gatekeeper, this provided hospitals with a mechanism for accountability, and resulted in lower rates of IOL. The ability of the gatekeeper to question an obstetrician’s decision-making and to ensure accountability for the decision was dependent on implicit or explicit senior management staff support. Effective gatekeepers were able to question the validity of the indication for IOL, and had close and supportive relationships with both midwifery and medical senior management of the hospital. Examples of senior management support include regular visibility at departmental clinical meetings and encouragement to develop leadership skills for the gatekeeper.

Strong gatekeepers often led to differences of opinion and conflict within the workplace with complaints between and within professional groups, despite health care practitioners aligning with their philosophical thoughts and practices of intervention in childbirth within the hospital. In one hospital, the two obstetricians:

‘*generally follow the principle that the less interference the better’* (B_P32_O).

which aligned with the beliefs of the midwives at that hospital. However, one participant perceived the challenging of inductions by the gatekeeper as part of:

*‘…an empire building process by the midwives….[there is a] very poor working relationship between the midwives, and there isn’t a very good working relationship between obstetricians and midwives’*(B_P32_O).

Although senior midwives, including midwifery managers were also in the gatekeeper role in hospitals with high rates of hospital IOL, they were unable to challenge or question the validity of the IOL due to the unit’s culture and structure of importance being elsewhere, for example one participant commenting that:

‘*a lot of the midwives want to drive policy change here, but unless [head of department] signs off on it, it doesn't happen’* (G_P25_O).

Accountability was a primary modifiable issue identified by participants in hospitals with high IOL rates to improve IOL decision-making in their hospitals. Some hospitals had considered or had begun to institute other ways of improving accountability, such as completing paper-based forms requiring an obstetrician to sign responsibility for the IOL decision.

In the only hospital with an administrative gatekeeper and a low rate of IOL, the low rates of IOL could be attributed to the attitudes of the obstetricians to avoid vaginal birth or IOL altogether and recommend CS for their women:

‘…*if everything's not optimal, I'd tend to...have a very low threshold to recommend a caesar[ean] rather than a - a vaginal delivery’* (D_P06_O).

## Discussion

We found that variations in decisions for IOL were based on the obstetrician’s perception of risk in the pregnancy which was influenced by the obstetrician’s personality and knowledge, their care relationship with the woman, how they involved the woman in decision-making, and resource availability. The role of a gatekeeper in the procedural aspects of arranging the IOL also influenced decision-making and therefore the hospital rate of IOL. Differences in IOL decision-making may assist in explaining the three fold difference in adjusted hospital IOL rates for nulliparae at term [13].

The most significant concern for obstetricians was fear of perinatal mortality, thus the desire to induce labour. Although previously neglected, perinatal mortality is on the international agenda (including the publication of 2011 and 2016 *Lancet* stillbirth series) [14, 15] and is goal three of the World Health Organisation Sustainable Development Goals [16]. Consistent with a Cochrane review, IOL has reduced perinatal mortality and morbidity for babies of pregnant women at or beyond 41 weeks gestation compared to expectant management [17, 18]. Therefore, some assert that offering IOL at a gestational age lower than 41 weeks will also reduce the perinatal mortality rate [19]. However, other studies have found an increase in IOL rate was not associated with any change in stillbirth rates, although these studies were underpowered for this rare outcome [20, 21]. Similarly, there is conflicting evidence from the United States when there was reduction in non-medically indicated IOL prior to 39 weeks gestation, with some studies finding no evidence of an increase in stillbirth [22, 23] and another study finding an increase in stillbirths [24]. Currently, a randomised controlled trial is in progress, investigating whether IOL for nulliparae at term will reduce adverse perinatal outcomes [25].

Variation in decision-making may relate to uncertainty and confusion in the literature about when to recommend IOL and maternal and perinatal consequences of IOL at that specific gestation for the particular indication of IOL. Apart from the concern of perinatal mortality leading to the desire to offer IOL, IOL had been traditionally thought to increase the risk of CS, but recent systematic reviews [26, 27] suggest otherwise. Therefore, there is less reluctance to offer IOL and currently there are ongoing debates whether IOL should be recommended at full term [19, 28]. Evidence from the general surgical literature has suggested much of everyday clinical practice is empirical; simply improving evidence does not lead to a reduction in variation in intervention rates and raises questions about the power of scientific evidence alone to reduce variation [29, 30]. Additionally, the effect of improving scientific evidence for interventions to reduce variation in hospital rates of the intervention (such as IOL) can be limited by the effect of patient preference.

Obstetricians and midwives acknowledged the importance of understanding women’s preferences for childbirth and their views in decision-making. Previous studies investigating women’s experiences of IOL found their main concerns were regarding lack of information and informed choice [11, 12, 31, 32]. Similarly, our study found that midwives questioned whether women were fully informed, describing the challenges of antenatal classes and time limitations in antenatal clinics. In discussing IOL with women, doctors concentrated more on the IOL process itself rather than on the risks of having or not having an IOL. Additionally, like another study, obstetricians describe the ‘framing effect’ [33] where information is selectively conveyed to women to influence women’s decision-making. Specific information brochures [31] or decision aids [34] may be a time efficient, transparent and cost-efficient way of communicating information about risks and benefits of IOL.

Accountability, demonstrated within the gatekeeper role and a supportive hospital culture, is potentially amenable to change. The role of the gatekeeper emphasises the importance of midwives in the care for pregnant women, having a different approach to maternity care, [35] with a less interventionalist attitude compared to obstetricians [36]. This study highlights the importance of effective leadership across organisations; a previous synthesis of qualitative work identified receptive and responsive senior management support was associated with high performance hospitals [37, 38]. The importance of accountability is closely related to ensuring patient safety. Studies into patient safety recommend empowerment by all staff to speak up, discuss concerns, mutual accountability and support without being put down [39]. Other forms of accountability have been implemented with success, such as a ‘hard stop approach’ to prevent non-medically indicated deliveries (i.e. elective deliveries), [40] but others assert that financial and regulatory changes may have unintended adverse effects [2] on outcomes.

Strengths of this study include a development of understanding of reasons for variation in decision-making for IOL that are not available in routinely collected data sets. Furthermore, through purposive sampling we were able to include a variety of clinical and midwifery staff in both rural and urban areas. A limitation of this study is that the choice of hospital was based on hospital IOL rates for nulliparae at term rather than the overall hospital IOL rate. However, previous work identified that most unexplained variation in hospital IOL rates occurred among nulliparae at term [9]. Additionally, the actual hospital IOL rate may have changed from 2011 to 2015, with some participants in hospitals describing institutional changes in response to a recognised high IOL rate in 2011. Another limitation of this study is that interviews explored opinions and beliefs rather than actual practice of the health care practitioner, and future research is planned to explore how obstetricians discuss decisions and recommendations for delivery with pregnant women. Social desirability bias may have influenced the responses of the participants that knew the researcher, but there were frank, differing opinions expressed in the interviews.

## Conclusion

Clinical decision-making for IOL is based on an obstetrician’s perception of risk of adverse perinatal outcomes in the pregnancy, their care relationship with the woman, how they involved the woman in decision-making, and resource availability. Improved communication, standardised risk assessment and accountability for IOL offer potential for reducing variation in IOL rates.

## Abbreviations

CS: Caesarean section
IOL: Induction of labour
NSW: New South Wales
